# Effect of temperature on the activity of efflux pumps in selected species of human opportunistic bacterial pathogens

**DOI:** 10.1590/0074-02760240162

**Published:** 2025-03-17

**Authors:** Dusan Hurton, Lukás Hleba, Jana Petrová, Maros Laho, Ján Koren, Adriana Liptáková

**Affiliations:** 1 Slovak University of Agriculture Nitra, Faculty of Biotechnology and Food Science, Department of Microbiology, Slovakia; 2 Comenius University Bratislava, Faculty of Medicine, Institute of Microbiology, Bratislava, Slovakia; 3 Slovak Academy of Sciences, Institute of Chemistry, Bratislava, Slovakia

**Keywords:** antibiotic resistance, efflux pumps, temperature

## Abstract

**BACKGROUND:**

Efflux pumps represents one of the most important mechanisms of antibiotic resistance. They allow bacteria to expel antibiotics from their cells before they reach the target site.

**OBJECTIVES:**

The main objective of this work was to examine how cultivation temperature and its variations affect the activity of efflux pumps in *Acinetobacter junii*, *Bacillus cereus*, and *Enterobacter cloacae* isolated from a skin swab.

**METHODS:**

The isolation and purification of bacterial colonies were done through the streak plate method. For the identification of bacterial species, MALDI-TOF was utilised. To detect the activity of efflux pumps, agar-ethidium bromide cartwheel method was implemented.

**FINDINGS:**

The accumulation of ethidium bromide (EtBr) in bacterial cells was higher at 43ºC than at 30ºC, so the activity of efflux pumps was reduced at 43ºC in all isolates. A temperature of 7ºC also caused increased cumulation of EtBr in the cells, hence decreasing the activity of efflux pumps in isolated bacteria. Moreover, *B. cereus* was more sensitive to meropenem at 43ºC than at 36ºC.

**MAIN CONCLUSIONS:**

The activity of efflux pumps and antibiotic resistance can be strongly affected by changes in incubation temperature *in vitro* in tested human opportunistic bacterial pathogens.

Antimicrobial resistance is one of the most serious concerns, not only in human medicine but also in animal production. It was estimated that antibiotic resistance will cause cumulative losses of trillions of dollars by the year 2050. Besides, the problem of antibiotic resistance in some strains of bacteria may be so problematic that new alternative therapeutic drugs need to be analysed.^1^ ^,^ ^2^ ^,^ ^3^

There are many mechanisms that bacteria use as a line of defence against antibiotics, namely, enzymatic inactivation, for instance by the activity of ß-lactamases, efflux, modification/protection of antibiotic target site, antibiotic sequestration, alteration of the cellular permeability, and target bypass. In this research, we focused mainly on the efflux mechanism based on efflux pumps. Efflux pumps are proteins integrated into the membrane of bacteria that serve as translocators promoting the excretion of antibiotics (for instance, ß-lactams, fluoroquinolones, tetracyclines), dyes, and other undesirable or toxic substances from bacterial cells. Several different families of efflux pumps are recognised: ATB-binding cassette (ABC), multidrug and toxic compound extrusion family (MATE), major facilitator superfamily (MFS), proteobacterial antimicrobial efflux family (PACE), resistance-nodulation cell division family (RND) and small multidrug resistance family (SMR). In gram-positive bacteria, efflux pumps consist of a single component. But in gram-negative bacteria, efflux pumps are composed of three parts: inner membrane transporter, periplasmic protein adapters, and outer membrane channel proteins. If a specific antibiotic can act as a substrate for some efflux pumps, the process of excreting can lead to a reduced antibiotic effect. However, not every antibiotic can be eliminated from bacterial cells by efflux pumps, for example, a semisynthetic tetracycline antibiotic, omadacycline, is resistant to some classes of efflux pumps but MexAB-OprM pump, which belongs to the resistance-nodulation-cell division superfamily (RND) can still eliminate omadacycline from bacterial cells.^4^ ^-^ ^12^

The activity of efflux pumps in the cell is not constant but can be modified according to environmental factors, such as antibiotics. In this case, antibiotic presence may lead to the overexpression of efflux pump genes, causing decreased sensitivity to antibiotics. But temperature may also play a role in this process because temperature, as a main environmental factor, has an impact on many biochemical processes in the bacterial cell, including bacterial efflux.^8^ ^,^ ^13^ ^,^ ^14^ For example, Martins et al. conducted an experiment that nicely illustrated the activity of efflux pumps in numerous bacterial species using EtBr, such as *Enterobacter* spp., *Escherichia* spp., or *Salmonella* spp., at cultivation temperatures of 4ºC and 37ºC.^13^ Another experiment that depicted the apparent influence of different cultivation temperatures (10ºC, 28ºC, and 35ºC) on efflux pumps in *Pseudomonas fluorescens* was performed by Adebusuyi et al., where radiolabelled phenanthrene was used instead of EtBr.^15^ Also, research done by Spaniol et al. showed how variations in incubation temperature may affect the activity of efflux pumps in *Moraxella catarrhalis*. In this case, bacteria were cultivated at temperatures of 26ºC and 37ºC, and as a substrate for efflux pumps, fluorescent dye bisbenzimid (Hoechst 33342) was utilised during the experiment.^16^

The purpose of this work was to analyse the effect of variations in an incubation temperature on efflux pumps of three human opportunistic pathogens (*Bacillus cereus*, *Enterobacter cloacae*, and *Acinetobacter junii*), which can cause skin and soft tissue infection, sometimes even in immunocompetent patients. Although *A. junii* is a rare pathogen, mainly in neonates, its true incidence in skin and soft tissue infections may be underestimated because phenotypical identification is complicated, and molecular diagnostics methods are needed.^17^ ^,^ ^18^ ^,^ ^19^ ^,^ ^20^ ^,^ ^21^

## MATERIALS AND METHODS

Bacterial strains were isolated from the surface of the human volunteer’s skin using a sterile swab. The swab was immersed in 5 mL of physiologic solution and mixed thoroughly. Subsequently, 500 µL of inoculum was spread onto Petri dishes containing MacConkey agar and meat extract B agar (both Himedia, India) using a glass spreader. Afterward, plates were incubated overnight. MacConkey agar was incubated at 30ºC and meat extract B agar at 37ºC. Then, we prepared pure cultures from grown colonies through the streaking cultivation method. After that, solitary colonies were submitted to mass spectrophotometry identification using MALDI-TOF Microflex LT with the software flexControl in version 3.4 (Bruker Daltonics, Germany).

We used two related strains of *B. cereus*: meropenem-sensitive (M1), isolated from human skin, and meropenem-resistant (M1-R), prepared in the laboratory. We induced resistance to meropenem in the second strain *in vitro* by cultivating the susceptible strain of *B. cereus* with a 10 µg meropenem disc for 20 h on Müeller-Hinton agar (MH) at 36ºC. One of the colonies growing inside the inhibition zone of the meropenem disc was extracted and cultivated overnight on fresh meat extract B agar. Resistance to meropenem was confirmed with the disc diffusion method according to EUCAST standards.^22^ We also carried out a disc diffusion method for antibacterial testing for the meropenem resistance of *B. cereus* at a cultivation temperature of 30ºC and 43ºC to determine if there would be any changes in the size of the inhibition zones.

The agar cartwheel method for testing the activity of efflux pumps was implemented using a Trypticase soy agar (TSA) plate with 0 mg/L (control), 1.5 mg/L, and 2.5 mg/L of EtBr. An inoculum of 0.5 McFarland was prepared from the cultures of acquired bacterial isolates and smeared on TSA agar using a sterile swab by one straight movement leading from the centre of the plate to the border. Afterward, we incubated the plates at 30ºC and 43ºC for 24 h. One of the tested isolates, *A. junii*, needed to be pre-cultivated at 37ºC for 18 h before cultivation at 43ºC due to poor growth at 43ºC without the pre-cultivation. The plates first cultivated at 30ºC continued to be incubated at 7ºC for 24 h.^13^ For a fluorescence visualisation of EtBr incorporated in colonies, a UV transiluminator (Bio-Rad, USA) was used, and the captured photos were compared to each other based on the recorded fluorescence intensity using ImageJ software (version 1.54g, NIH, USA).

## RESULTS

MALDI-TOF confirmed the isolation of three species of opportunistic bacterial pathogens from the swab sample: *B. cereus*, *E. cloacae, and A. junii,* with an average score of 2.1 (default double testing).

Regarding results from the agar cartwheel method, we found that temperature can affect the efflux of EtBr from the bacterial cells, but with different intensities in the isolates.

**Fig. 1:**
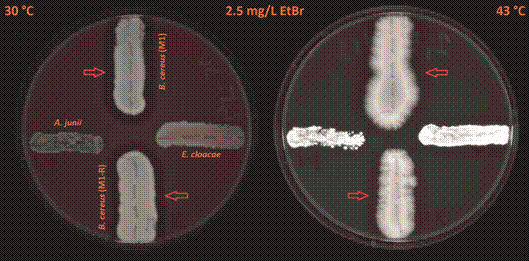
an accumulation of EtBr expressed by different strengths of fluorescence in the tested species of human opportunistic bacterial pathogens at 30ºC and 43ºC in *Acinetobacter junii* (west), *Enterobacter cloacae* (east), meropenem-sensitive strain of *Bacillus cereus* (M1; north), and meropenem-resistant strain of *B. cereus* (M1-R; south) on Trypticase soy agar plates containing 2.5 mg/L of EtBr. Red arrows indicate clear zones (an area of reduced fluorescence) around growing colonies of *B. cereus* strains.

**Fig. 2:**
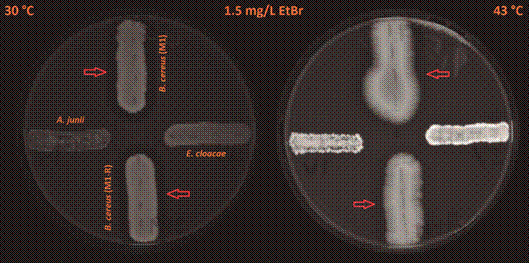
an accumulation of EtBr expressed by the various strengths of fluorescence in the tested species of human opportunistic bacterial pathogens at 30ºC and 43ºC in *Acinetobacter junii* (west), *Enterobacter cloacae* (east), meropenem-sensitive strain of *Bacillus cereus* (M1; north), and meropenem-resistant strain of *B. cereus* (M1-R; south) on Trypticase soy agar plates containing 1.5 mg/L of EtBr. Red arrows indicate clear zones (an area of reduced fluorescence) around growing colonies of *B. cereus* strains.

When comparing the cumulation of EtBr in the bacterial colonies of *A. junii* and *E. cloacae*, there was a 3.3-5.4-fold stronger fluorescence signal at a cultivation temperature of 43ºC than at 30ºC with both EtBr concentrations used (2.5 mg/L and 1.5 mg/L) (Figs 1-2). These data suggest impaired efflux of EtBr out of the bacterial cells at a cultivation temperature of 43ºC. When comparing colonies of *B. cereus*, both strains M1 and M1-R (meropenem-sensitive and meropenem-resistant) exhibited similar strength of the fluorescence signal (only a 1.4-1.7-fold stronger fluorescence signal at a cultivation temperature of 43ºC than 30ºC) with both EtBr concentrations used (Figs 1-2).

**Fig. 3:**
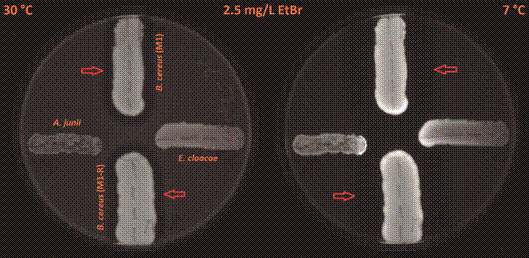
an accumulation of EtBr expressed by the various strengths of fluorescence in the tested species of human opportunistic bacterial pathogens at 30ºC and 7ºC in *Acinetobacter junii* (west), *Enterobacter cloacae* (east), meropenem-sensitive strain of *Bacillus cereus* (M1; north), and meropenem-resistant strain of *B. cereus* (M1-R; south) on Trypticase soy agar plates containing 2.5 mg/L of EtBr. The plates were first incubated at 30ºC for 24 h. Subsequently, the plates continued to be cultivated at 7ºC for another 24 h. Red arrows indicate clear zones (an area of reduced fluorescence) around growing colonies of *B. cereus* strains.

**Fig. 4:**
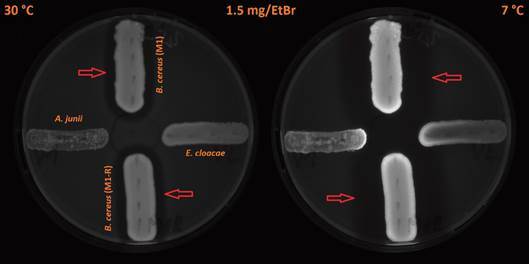
an accumulation of EtBr expressed by the various strengths of fluorescence in the tested species of human opportunistic bacterial pathogens at 30ºC and 7ºC in *Acinetobacter junii* (west), *Enterobacter cloacae* (east), meropenem-sensitive strain of *Bacillus cereus* (M1; north), and meropenem-resistant strain of *B. cereus* (M1-R; south) on Trypticase soy agar plates containing 1.5 mg/L of EtBr. The plates were first incubated at 30ºC for 24 h. Subsequently, the plates continued to be cultivated at 7**º**C for another 24 h. Red arrows indicate clear zones (an area of reduced fluorescence) around growing colonies of *B. cereus* strains.

**Fig. 5:**
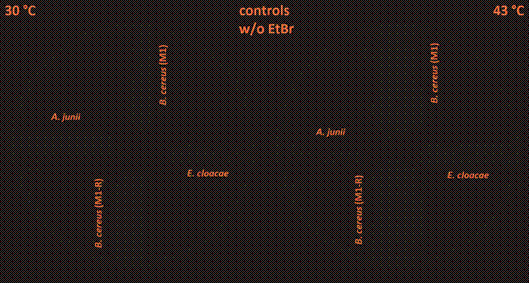
the control plates with the isolates were cultivated at 30ºC and 43ºC without any added EtBr to the medium (Trypticase soy agar).

When we cooled down the plates cultivated at a temperature of 30ºC to a temperature of 7ºC and kept them at this temperature for a period of 24 h, changes in the fluorescence signals of the colonies occurred: a 1,4-2.2-fold increase in *B. cereus* strains, a 2.5-fold increase in *E. cloacae*, and a 1.2-1.5-fold increase in *A. junii*. The changes were visible mainly on the borders of the colonies (Figs 3-4). This finding indicates that there was increased cumulation of EtBr in the bacterial cells at 7ºC. Hence, the activity of efflux pumps was diminished at that temperature. We also compared the intensity of the fluorescence signal of the meropenem-resistant strain of *B. cereus* (M1) with that of the meropenem-sensitive strain of *B. cereus* (M1-R). We found out that there was no substantial difference in the strength of the fluorescence signal at any temperature (differences in the range between 1.04 and 1.21-fold). We also cultivated control plates without EtBr (0 mg/L) at 30ºC and 43ºC (Fig. 5); all fluorescence readings are provided in Tables I-II.

**TABLE I:** Fluorescence values

	2.5 mg/L EtBr			1.5 mg/L EtBr			w/o EtBr		
	30**º**C	43**º**C	7**º**C	30**º**C	43**º**C	7**º**C	30**º**C	43**º**C	7**º**C
*Acinetobacter junii*	34.2	170.6	86.0	23.9	129.5	58.9		7.2	
*Enterobacter cloacae*	56.4	188.3	83.4	37.0	154.3	42.9		7.3	
*Bacillus cereus* M1	91.8	136.5	148.7	56.9	97.8	123.6		7.4	
*Bacillus cereus* M1-R	100.7	144.1	137.4	64.5	101.6	115.7		7.2	

Fluorescence readings of the plates incubated at 7ºC, 30ºC, and 43ºC were acquired by using ImageJ software (version 1.54g, NIH, USA).

**TABLE II:** Fold change of fluorescence values

	2.5 mg/L EtBr			1.5 mg/L EtBr		
	30**º**C	43**º**C	7**º**C	30**º**C	43**º**C	7**º**C
*Acinetobacter junii*	1.0	4.99	2.51	1.0	5.42	2.46
*Enterobacter cloacae*	1.0	3.34	1.48	1.0	4.17	1.16
*Bacillus cereus* M1	1.0	1.49	1.62	1.0	1.72	2.17
*Bacillus cereus* M1-R	1.0	1.43	1.36	1.0	1.58	1.79

Comparison of fluorescence readings from the plates incubated at 43ºC and 7ºC with fluorescence readings from the plates incubated at 30ºC obtained by using ImageJ software (version 1.54g, NIH, USA).

We also observed clear zones (areas of reduced fluorescence) around the growing colonies of *B. cereus* (Figs 1-2; these zones are indicated by the arrows). We found no similar clear zones around *A. junii* or *E. cloacae*. Continued cultivation at an incubation temperature of 7ºC expanded these zones, compared to a cultivation temperature of 30ºC (Figs 3-4; zones of reduced fluorescence are indicated by the arrows).

**Fig. 6:**
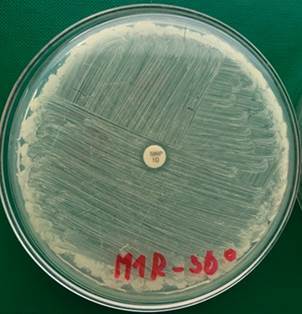
a meropenem-resistant strain of *Bacillus cereus* (M1-R) on Müeller-Hinton agar plate with a 10 µg meropenem disc after 20 h at 36ºC. The diameter of the inhibition zone was 8 mm.

The disc diffusion method confirmed resistance to meropenem in the M1-R strain (an original 27 mm inhibition zone reduced to 8 mm) at a cultivation temperature of 36ºC (Fig. 6).

**Fig. 7:**
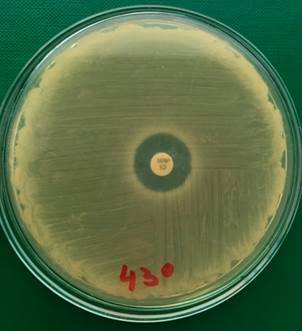
a meropenem-resistant strain of *Bacillus cereus* (M1-R) on Müeller-Hinton agar plate with a 10 µg meropenem disc after 20 h at 43ºC. The diameter of the inhibition zone was 16 mm.

We also executed the disc diffusion method at a cultivation temperature of 30ºC and 43ºC for both strains of *B. cereus* (M1 and M1-R). We experimentally discovered that M1 and M1-R strains cultivated on MH agar plates were more sensitive to meropenem at a cultivation temperature of 43ºC than 36ºC or 30ºC. An inhibition zone around the meropenem disc increased from 8 mm to 16 mm in the M1-R strain at 43ºC (Fig. 7).

**Fig. 8:**
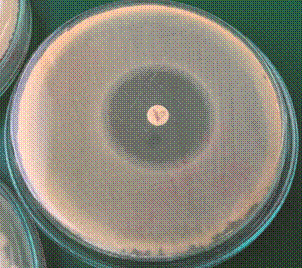
a meropenem-sensitive strain of *Bacillus cereus* (M1) on Müeller-Hinton agar plate with a 10 µg meropenem disc after 20 h at 43ºC. The diameter of the inhibition zone was 31 mm. No resistant colonies of *B. cereus* grew up inside the inhibition zone.

**Fig. 9:**
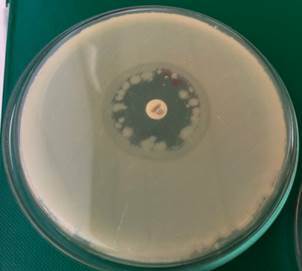
a meropenem-sensitive strain of *Bacillus cereus* (M1) on Müeller-Hinton agar plate with a 10 µg meropenem disc after 20 h at 36ºC. The diameter of the inhibition zone was 27 mm. Many resistant colonies of *B. cereus* (~ 34) grew up inside the inhibition zone.

**Fig. 10:**
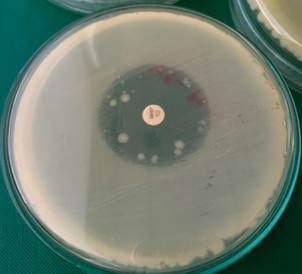
a meropenem-sensitive strain of *Bacillus cereus* (M1) on Müeller-Hinton agar plate with a 10 µg meropenem disc after 20 h at 30ºC. The diameter of the inhibition zone was 29 mm. Several resistant colonies of *B. cereus* grew up inside the inhibition zone (~ 2-fold less than at 36ºC).

In the M1 strain, there was an increase from 27 mm to 31 mm at 43ºC (Fig. 8); we repeated the experiments several times to exclude coincidence. We also found out that it was not possible to induce resistance to meropenem in meropenem-sensitive strain (M1) at an incubation temperature of 43ºC (Fig. 8) compared to an incubation temperature of 30ºC (Fig. 9) and 36ºC (Fig. 10).

## DISCUSSION

Ethidium bromide is a well-known chemical in molecular laboratories, mainly used as a staining dye for DNA visualisation for polymerase chain reaction (PCR) analyses. Interestingly, this compound may also serve as a substrate for efflux pumps in various species of bacteria and provide a sophisticated yet cheap method for visualisation of the activity of efflux pumps. The mechanism behind this method is that free EtBr is only slightly fluorescent, but when it binds to DNA, there is a 25-fold increase in the strength of the fluorescent signal. When it binds to RNA, there is a 21-fold enhancement in the fluorescence intensity.^23^ However, there were some concerns that EtBr should not be used anymore or replaced by other dyes due to its toxicity/mutagenicity but these worries might be exaggerated. Also, some replacements of EtBr showed even higher acute toxicity than EtBr itself.^24^ ^,^ ^25^

Carbapenemases are usually responsible for resistance to meropenem. However, meropenem may also serve as the substrate for efflux pumps in different species of bacteria.^8^ That was the reason why we used meropenem-sensitive (M1) and meropenem-resistant (M1-R) strains of *B. cereus* in our experiment: to examine if there would be a difference in the cumulation of EtBr, hence the difference in the activity of efflux pumps between these two strains.

Although observation of the activity of efflux pumps by using EtBr has already been done in the past, there is still room for experimentation with this method with different strains of bacteria and at different temperatures. We used cultivation temperatures of 7ºC, 30ºC, and 43ºC. We used 7ºC because, in previous works, temperatures of 4ºC and 10ºC were used so we opted for the middle value. We chose 30ºC because many bacterial species have an optimal growth temperature of 30ºC, and we picked a temperature of 43ºC because it is the highest temperature a human skin can tolerate without restrictions in blood flow.^26^ We found that these cultivation temperatures plausibly depicted how different cultivation temperatures may affect the cumulation of EtBr in the cells of isolated human opportunistic bacteria, thus the activity of their efflux pumps (a 1.4-5.4-fold stronger fluorescence signal at 43ºC than at 30ºC). At a temperature of 7ºC, the increase in the fluorescence signal was not so dramatic (only a 1.2-2.5-fold stronger fluorescence signal at 7ºC than at 30ºC). According to these results, cold shock had a weaker inhibition effect on the activity of efflux pumps than heat shock in *E. cloacae* and *A. junii* but not in *B. cereus*, where the impact of both temperatures (7ºC and 43ºC) was very similar for both strains (1.4-1.7-fold increase for cold shock versus 1.4-2.2-fold increase for heat shock in the strength of fluorescence signal). Adebusayi et al. found that efflux pumps in *P. fluorescens* had higher output at a cultivation temperature of 10ºC compared to 28ºC or 35ºC.^15^ However, Martins et al. conclude that *Salmonella* spp., *Enterobacter* spp., and *Escherichia* spp. have reduced activity of efflux pumps at a cultivation temperature of 4ºC compared to a cultivation temperature of 37ºC.^13^ If we take these and our results into some context, relatively lower temperatures may increase the activity of efflux pumps in some strains and decrease it in others, suggesting this process may be specific to different species of bacteria.

Regarding the clear zones we mentioned in the results, it is likely that *B. cereus* is capable of metabolising EtBr. Sukhumungoon et al. made an experiment that showed that *Bacillus thuringiensis* can degrade EtBr. *B. thuringiensis* strains competent to disintegrate EtBr had very similar clear zones around colonies as *B. cereus* had in our experiment (both strains; M1 and M1-R).^27^ This finding indicates that *B. cereus* can also degrade EtBr. This observation could probably also explain why colonies of *B. cereus* had a weaker fluorescence signal, even at 43ºC compared with *A. junii* or *E. cloacae* cultivated at the same temperature. However, these zones of reduced fluorescence are weaker at 43ºC. An explanation for this observation could be that *B. cereus* metabolised EtBr at a slower rate at 43ºC compared with 30ºC or 7ºC.

In 2020, Khan conducted research in which he showed how relatively higher temperatures and residues of antibiotics (from river water) can act synergistically in the process of inducing antibiotic resistance. This work emphasised that temperature might be an even more significant factor for the induction of antibiotic resistance than the residues of antibiotics themselves. At a temperature of 17ºC, Khan could not decrease sensitivity to ceftazidime in *Klebsiella oxytoca*, even after 600 generations. At a cultivation temperature of 25ºC, it was possible to do that after only 200 generations, and after 600 generations, there was an 8 mm reduction in the inhibition zone of ceftazidime. Similar results were observed for meropenem.^28^ Similarly, during our research, we tried to induce resistance to meropenem in the meropenem-sensitive strain of *B. cereus* (M1)*.* We also tried to determine if a relatively higher temperature may influence the process itself. We induced resistance to meropenem at an incubation temperature of 30ºC and 36ºC. Resistant colonies were slightly bigger at an incubation temperature of 36ºC, and there were more of them. Interestingly, it was not possible to produce any resistant colonies at a cultivation temperature of 43ºC. These results indicate that relatively high temperatures (43ºC) could cause bacteria to regain their antibiotic sensitivity to some extent. However, Verdugo-Rodríguez et al. were able to induce resistance to rifampicin in the antibiotic-free environment during thermal stress (at a temperature of 42ºC) in *Escherichia coli* after 2000 generations.^29^ This could indicate that the effect of relatively higher temperatures might depend on an antibiotic type or even on the presence of the antibiotic itself.

According to Cruz-Loya et al., some antibiotics can have a synergic effect with cold or hot temperatures. For instance, ß-lactams and thermal stress may act synergistically; specifically, thermal stress can increase the permeability of the bacterial membrane and decrease the efflux of antibiotics from the bacterial cell.^30^ This claim could plausibly explain why the meropenem-resistant strain of *B. cereus* (M1-R) was more sensitive to meropenem at a relatively higher temperature of 43ºC, and it could also explain why we were not able to acquire resistant colonies of *B. cereus* at an incubation temperature of 43ºC in the presence of meropenem.

Based on the results, we suggest that applying heat to the skin wound infected with certain bacterial strains during the antibiotic treatment (topical or systemic) could enhance the antibiotic effect and decrease the healing time of the infection site. According to Wienert et al., human skin can tolerate a temperature of 43ºC for 8 h without a decrease in blood flow.^26^ Besides that, according to Khan et al., using heat on wounds can improve the innate immunity of the wound site and can also enhance the healing rate of the wound.^31^
